# A miR172e/*TOE3* Module from the Halophyte *Halostachys caspica* Regulates Plant Multiple Abiotic Stress Tolerance via Cellular Homeostasis

**DOI:** 10.3390/plants15071087

**Published:** 2026-04-01

**Authors:** Yadi Wang, Jieyun Ji, Youling Zeng

**Affiliations:** Xinjiang Key Laboratory of Biological Resources and Genetic Engineering, College of Life Science and Technology, Xinjiang University, Urumqi 830017, China; 107556524190@stu.xju.edu.cn (Y.W.); jjy960922@outlook.com (J.J.)

**Keywords:** *Halostachys caspica*, the HcmiR172e/*HcTOE3* module, abiotic stresses, transgenic *Arabidopsis*

## Abstract

Salt, drought and freezing stress were major abiotic factors limiting plant growth, development and yield. *Halostachys caspica* (Amaranthaceae), a halophyte native to saline-arid desert regions, tolerated multiple abiotic stresses, but its molecular mechanisms of stress tolerance remain unclear. By integrating the small RNA library and transcriptome data of *H. caspica* under high salinity, HcmiR172e was identified as a differentially expressed miRNA and selected for the study of multiple abiotic stress responses. Using its mature sequence (20 nt) to align with upregulated genes from the transcriptome, *HcTOE3* (AP2 subfamily transcription factor belonging to the AP2/ERF family) was preliminarily predicted as its target gene through bioinformatic analysis. Our previous work demonstrated that *HcTOE3* was strongly upregulated by multiple abiotic stresses, including salinity, drought, heat and low temperature. Furthermore, overexpression of *HcTOE3* conferred freezing tolerance to *Arabidopsis* throughout the entire growth period. In this study, miRNA expression analyses showed that HcmiR172e was significantly downregulated in the assimilating branches of *H. caspica* under low temperature, heat, salt, drought, oxidative stress and abscisic acid (ABA) application. Tobacco transient expression assays and 5′RLM-RACE confirmed that HcmiR172e directly cleaved *HcTOE3* transcripts in the region close to the 5′end of the ORF. HcmiR172e-overexpressing *Arabidopsis* displayed increased sensitivity to salt, drought, freezing stresses and ABA treatment, along with enhanced growth inhibition, elevated reactive oxygen species (ROS) accumulation, decreased osmolyte content and downregulation of stress-responsive genes. In contrast, *HcTOE3*-overexpressing *Arabidopsis* exhibited the opposite phenotypes, physiological responses and corresponding gene expression patterns under multiple stress treatments. These findings collectively elucidated the antagonistic regulatory roles of HcmiR172e and HcTOE3 in plant abiotic stress responses, providing novel molecular targets for engineering stress-tolerant crops for saline, arid, freezing environments.

## 1. Introduction

Abiotic stresses were major environmental constraints severely impacting plant growth, development and yield [1]. When plants were exposed to salinity, drought and extreme temperatures, they suffered osmotic stress and ion toxicity, which perturbed the cellular oxidative balance, inducing the excessive accumulation of reactive oxygen species (ROS) [2]. Stress-induced increases in ROS could, on one hand, induce gene expression that restored the equilibrium between intracellular and intercellular redox signalling [3]; on the other, when accumulated excessively, these cytotoxic molecules could initiate lipid peroxidation cascades and compromise membrane integrity [4]. To mitigate these adverse effects, plants have developed diverse adaptation mechanisms encompassing morphological adjustments, physiological responses, and molecular regulation involving stress-responsive genes [5]. Among these regulatory mechanisms, microRNAs (miRNAs) have been recognized as crucial players in plant stress adaptation [6].

MicroRNAs (miRNAs) were a class of endogenous small non-coding RNAs (19–25 nucleotides in length) that have emerged as key post-transcriptional regulators involved in plant biological processes by binding to complementary sequences of target mRNAs, leading to translational repression or transcript degradation [7,8]. Recently, an increasing number of miRNAs have been reported to function in plant abiotic stress [9]. For instance, miR169q was induced under salt stress, negatively regulating their target genes NF-YA and ERF to modulate root and leaf growth in sweet potatoes, thereby enhancing salt tolerance [10]. In poplar, miR6445 negatively regulated the expression of *PtrGSTU23* by targeting the transcription factor *PtrNAC029*, forming a ROS-stable core module that improved drought resistance [11]. In sugarcane, miR319 was found to upregulate and cleave the target genes *TCP-PCF6* and *GAMyb* under cold stress, reducing energy consumption and delaying growth, thereby enhancing plant adaptation to low temperature [12]. Among the diverse miRNAs, miR172 has been characterized as a conserved player in regulating plant development and flowering time by targeting AP2/ERF transcription factor and AP2-like genes such as *TARGET OF EAT 1/2/3 (TOE)*, *SCHLAFMUTZE (SMZ)* and *SCHNARCHZAPFEN (SNZ)* [13]; emerging evidence also suggests its involvement in stress responses as well [14]. The mature miR172 sequence was 20 nucleotides in length, although the number of mature and precursor miRNAs varies among plant species. For example, there were five miR172 loci each in maize [15], *Arabidopsis* [16], potato [17], while rice had four miR172 loci [18]. In *H. caspica*, five HcmiR172 members were identified which HcmiR172e was significantly downregulated under salt stress [19]; however, its target gene in *H. caspica* remains unknown.

As the main targets of miR172, the APETALA2/Ethylene (AP2/ERF) transcription factors constituted one of the largest transcription factor families in plants and were involved in diverse biological processes, including growth, development, and stress responses [20,21,22]. In line with the emerging role of miR172 in abiotic stress, increasing evidence indicated that miR172 modulated stress tolerance across multiple species by targeting members of the AP2 subfamily: in cereal crops, the miR172s/*IDS1* (AP2 subfamily) regulatory module was found to function as a crucial molecular rheostat in maintaining ROS homeostasis during salt stress, mainly by balancing the expression of a group of ROS-scavenging genes, thereby enhancing salt tolerance [18]; in tobacco, following TMV infection, miR172 was found to be significantly enriched in infected cortical tissues (ICTs), leading to reduced abundance of the target gene *TOE3* and subsequent inhibition of defense-related gene expression, thereby facilitating higher viral accumulation in ICTs and negatively regulating disease resistance [23]; In peony, PomiR172d was found to negatively correlate with its target gene *PoARR*, and overexpression of *PoARR* was further confirmed to enhance ROS scavenging to maintain homeostasis, thereby improving plant resistance to drought stress [24]. Nevertheless, how HcmiR172e participates in abiotic stress responses through regulating its target gene in *H. caspica* remains unclear.

*H. caspica*, a halophyte native to the semi-desert regions of Central Asia, exhibits strong stress tolerance [25]. We previously identified *HcTOE3*, an AP2 subfamily transcription factor highly expressed under multiple abiotic stresses, and showed that its overexpression enhances freezing tolerance in *Arabidopsis* by activating cold-responsive genes [26]. Bioinformatics analysis and sequence conservation revealed *HcTOE3* as a predicted target of HcmiR172e, consistent with the conserved miR172-target regulatory module in plants [27,28,29]. Although *HcTOE3* was previously implicated in freezing tolerance, whether it acts downstream of HcmiR172e and whether the HcmiR172e/*HcTOE3* module functions in plant responses to salt, drought, and freezing stress remained unclear. Whether the HcmiR172e/*HcTOE3* module functioned in plant responses to salt, drought and freezing stress remained unclear. The present study aimed to verify the targeted cleavage relationship of *HcTOE3* by HcmiR172e in *H*. *caspica*, to dissect the abiotic stress functions of the HcmiR172e/*HcTOE3* module in transgenic *Arabidopsis* under salt, drought and low-temperature stresses, and to provide a theoretical basis for the identification and application of key stress-resistant genes in halophytes.

## 2. Results

### 2.1. Expression Pattern of HcmiR172e in H. caspica Under Abiotic Stresses and ABA Treatment

**Figure 1:**
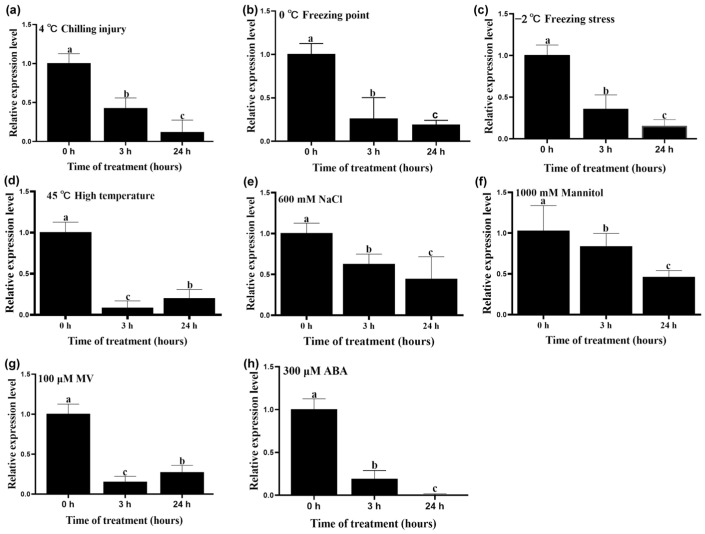
Expression profile of *H. caspica* miR172e in the *H. caspica* assimilating branches under different abiotic stresses and ABA treatment. Expression levels decreased under abiotic stress conditions. (**a**) −2 °C Freezing stress, (**b**) 0 °C Freezing point, (**c**) 4 °C Chilling injury, (**d**) 45 °C High temperature, (**e**) 600 mM NaCl, (**f**) 1000 mM Mannitol, (**g**) 100 μM MV, (**h**) 300 μM ABA. Reverse transcription-quantitative polymerase chain reaction analysis of miR172 expression in assimilating shoots. Data are shown as means ± SD (n = 3). Different letters above bars indicate significant differences at 0 h, 3 h, 24 h time points (*p* < 0.05, one-way ANOVA).

In the small RNA library constructed from *H. caspica* roots exposed to 600 mM NaCl stress, HcmiR172e was identified as significantly downregulated and selected as a candidate for further investigation [19]. Quantitative expression analysis subsequently revealed that HcmiR172e was markedly suppressed under multiple abiotic stresses, including low temperatures (4 °C, 0 °C, −2 °C), high temperature (45 °C), salt (600 mM NaCl), drought (1000 mM mannitol), oxidative stress (100 μM methyl viologen, MV) and exogenous ABA treatment (300 μM) (Figure 1a–h).

### 2.2. The Targeted Cleavage of HcTOE3 by HcmiR172e Through Experimental Methods

**Figure 2:**
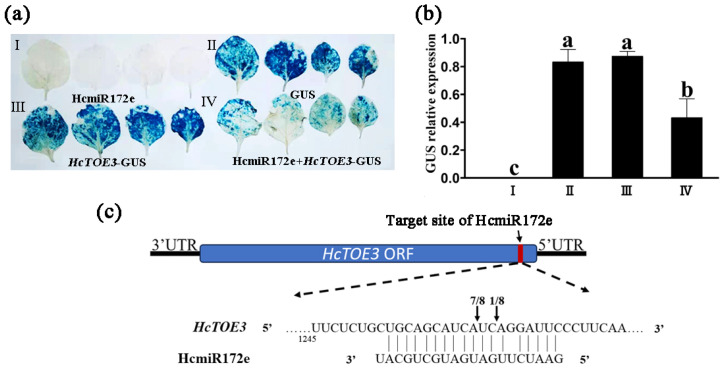
Validation of the targeting relationship between HcmiR172e and *HcTOE3*. (**a**) GUS histochemical staining of tobacco leaves transiently expressing different constructs. I, pCAMBIA2300-pre-HcmiR172e; II, pCAMBIA1304-GUS; III, pCAMBIA1304-*HcTOE3*-GUS; IV, pCAMBIA2300-pre-HcmiR172e and pCAMBIA1304-*HcTOE3*-GUS. (**b**) GUS relative expression in different groups. Data are shown as means ± SD (n = 3). (**c**) Schematic of HcmiR172e targeting site in *HcTOE3* and sequence alignment. The red block indicates the targeting site within *HcTOE3* ORF. 5′RLM-RACE results show 7/8 clones have cleavage at the predicted site. Different letters above bars indicate significant differences between samples (*p* < 0.05, one-way ANOVA).

To verify whether HcmiR172e exerts targeted regulation on *HcTOE3*, this study employed a tobacco transient expression system combined with 5′RLM-RACE technology. Analysis of the GUS gene by histochemical staining and relative expression revealed that, compared with *HcTOE3*-GUS expressed alone in tobacco leaves, co-expression of *HcTOE3*-GUS with HcmiR172e significantly reduced GUS expression. No staining signal was observed in leaves expressing HcmiR172e alone, transformed with the construct of pCAMBIA2300-pre-HcmiR172e, whereas leaves transformed with the empty vector (pCAMBIA1304-GUS) exhibited strong staining (Figure 2a,b), indicating that HcmiR172e mediated the cleavage and degradation of *HcTOE3*. 5′RLM-RACE further confirmed that HcmiR172e directed precise cleavage at the 1263 nt site within the *HcTOE3* ORF 3′end, with this cleavage site detected in 7 out of 8 clones (Figure 2c).

### 2.3. The HcmiR172e/HcTOE3 Module Regulating Salt and Drought Tolerance in Arabidopsis

**Figure 3:**
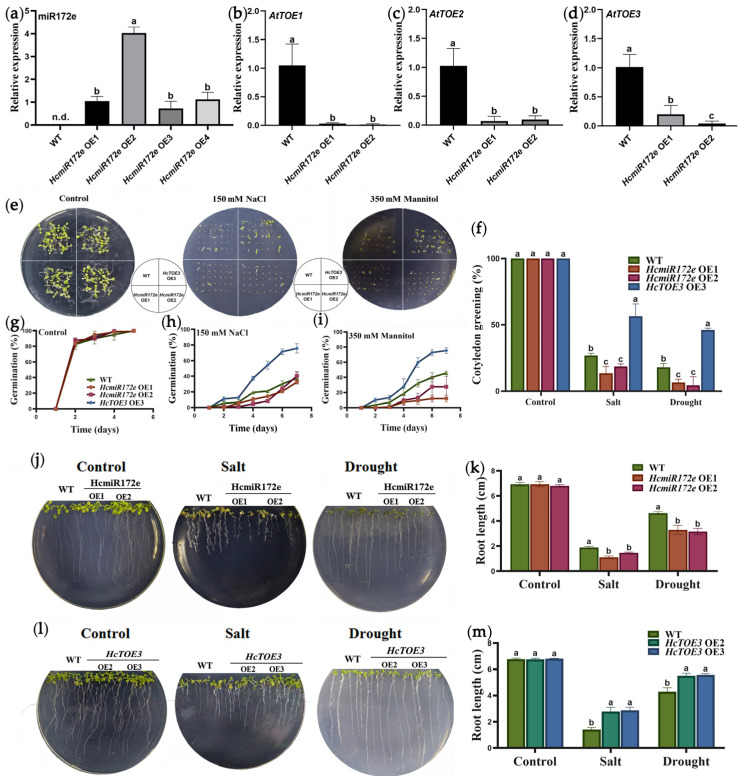
HcmiR172e and *HcTOE3* regulate seed germination and post-germination growth in *Arabidopsis* under salt and drought stresses. (**a**) Relative expression of mature miR172e in HcmiR172e-OE lines and WT. (**b**–**d**) Relative expression levels of (**b**) *AtTOE1*, (**c**) *AtTOE2* and (**d**) *AtTOE3* in *Arabidopsis* overexpressing HcmiR172e and WT. (**e**–**i**) Seed germination and cotyledon greening of different types of *Arabidopsis* under the control, 150 mM NaCl and 350 mM mannitol (drought stress) conditions. (**j**–**m**) Average root length under control, salt and drought stresses. (**j**,**k**) HcmiR172e-OE lines and (**l**,**m**) *HcTOE3*-OE lines under control, salt and drought stresses. Different letters above bars indicate significant differences among genotypes under salt and drought treatments (*p* < 0.05, one-way ANOVA).

To elucidate the regulatory role of the HcmiR172e/*HcTOE3* module in abiotic stress responses, single-copy homozygous transgenic *Arabidopsis* lines overexpressing HcmiR172e (35S:HcmiR172e, designated as high-expression lines HcmiR172e-OE1 and HcmiR172e-OE2) were generated (Figure 3a). The *HcTOE3*-overexpressing transgenic *Arabidopsis* lines (*HcTOE3*-OE2 and *HcTOE3*-OE3) were obtained from a previous study [26]. Expression analysis of potential endogenous target genes of miR172e (*AtTOE1*, *AtTOE2* and *AtTOE3*) in the HcmiR172e-OE lines revealed significant downregulation of all three genes (Figure 3b–d), indicating that HcmiR172e also mediated targeted cleavage of these genes in *Arabidopsis*.

Under non-stress conditions, no significant differences were observed in germination rate (all reaching 100% after 7 days), cotyledon greening rate and root length among all lines (Figure 3e,f,i). Under 150 mM NaCl stress, *HcTOE3*-OE3 outperformed WT, exhibiting higher germination (70% vs. 30%), cotyledon greening (60% vs. 25%) and radicle length (2.5 cm vs. 1.4 cm). In contrast, HcmiR172e-OE lines showed significantly lower values across all three parameters (25%, 15% and 0.8 cm, respectively) compared to WT. Similarly, under 350 mM mannitol-simulated drought stress, *HcTOE3*-OE3 again surpassed WT (70%/45%/5.5 cm vs. 40%/20%/4 cm), while HcmiR172e-OE lines exhibited reduced performance (25%/8%/2.5 cm) (Figure 3j–m). These results collectively indicated that HcmiR172e negatively regulated, whereas its target gene *HcTOE3* positively regulated, *Arabidopsis* tolerance to salt and drought stresses.

### 2.4. HcmiR172e/HcTOE3 Module Regulates Antioxidant and Osmotic Adjustment Capacities in Arabidopsis Under Salt and Drought Stress

To investigate the function of the HcmiR172e/*HcTOE3* module in antioxidant activity and osmotic adjustment under salt and drought stresses, ROS homeostasis, physiological parameters and stress-responsive gene expression were analyzed in transgenic *Arabidopsis* lines.

**Figure 4:**
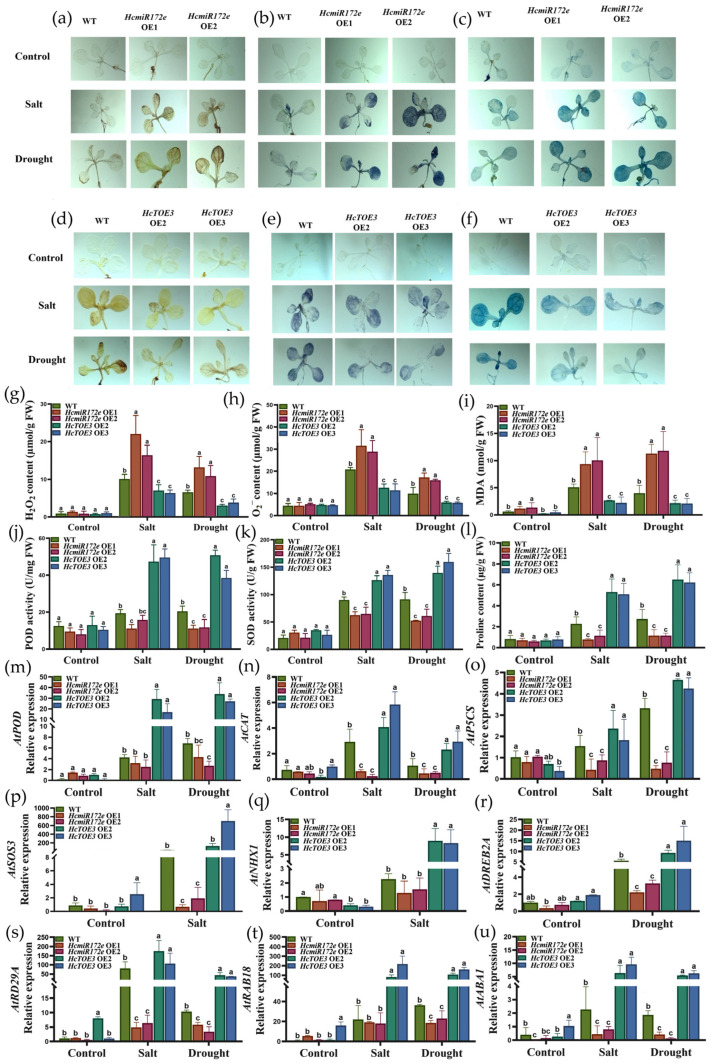
HcmiR172e and HcTOE3 oppositely modulate antioxidant defense and osmotic adjustment in *Arabidopsis* under salt and drought stresses. (**a**–**f**) Histochemical staining of 3-week-old *Arabidopsis* seedlings: DAB staining for H_2_O_2_ accumulation in (**a**) HcmiR172e-OE lines and (**d**) *HcTOE3*-OE lines; NBT staining for O^2•−^ accumulation in (**b**) HcmiR172e-OE lines and (**e**) *HcTOE3*-OE lines; Evans blue staining for cell death in (**c**) HcmiR172e-OE lines and (**f**) *HcTOE3*-OE lines under control, salt and drought conditions. (**g**–**i**) Quantification of (**g**) H_2_O_2_, (**h**) O^2•−^ and (**i**) MDA contents. (**j**–**l**) Activities of (**j**) POD, (**k**) SOD and (**l**) proline content. (**m**–**u**) Relative expression of antioxidant enzyme genes (**m**) *AtPOD* and (**n**) *AtCAT*, osmotic regulation gene (**o**) *AtP5CS*, and salt-drought stress responsive genes (**p**) *AtSOS3*, (**q**) *AtNHX1*, (**r**) *AtDREB2A* and (**s**) *AtRD29A* and ABA-related genes (**t**) *AtRAB18* and (**u**) *AtABA1* via qRT-PCR in *Arabidopsis* under corresponding stresses. Data are presented as means ± SD of three biological replicates. Different letters above bars indicate significant differences among genotypes under salt and drought treatments (*p* < 0.05, one-way ANOVA).

Environmental stresses trigger excessive ROS accumulation, leading to oxidative burst, membrane lipid peroxidation and ultimately cell death [30]. To visualize ROS dynamics and cellular damage, we performed histochemical staining on 3-week-old seedlings using DAB and NBT to detect H_2_O_2_ (Figure 4a,d) and O^2•−^ (Figure 4b,e), respectively, alongside Evans blue staining to assess membrane integrity and cell viability (Figure 4c,f). Under optimal growth conditions, WT, HcmiR172e-OE lines and *HcTOE3*-OE lines displayed basal ROS levels and membrane integrity. However, following salt and drought treatments, HcmiR172e-OE lines exhibited markedly intensified DAB and NBT staining, indicative of elevated H_2_O_2_; and O^2•−^ accumulation, concomitant with pronounced Evans blue staining reflecting exacerbated membrane damage and cell death (Figure 4a–f).

Quantitative validation through biochemical assays confirmed that HcmiR172e-OE lines accumulated significantly higher H_2_O_2_ and O^2•−^ contents, corroborating the histochemical observations (Figure 4g,h). Consistently, lipid peroxidation assessed by Malondialdehyde (MDA) content revealed that HcmiR172e-OE lines suffered aggravated oxidative damage, whereas *HcTOE3*-OE lines demonstrated enhanced membrane stability with reduced MDA levels under stress (Figure 4i).

Subsequently, the antioxidant capacity and osmotic adjustment potential were evaluated in these transgenic lines. Under non-stress conditions, no significant differences were observed in POD and SOD activities, or proline content among three different types of *Arabidopsis* (Figure 4j–l). Strikingly, upon salt and drought exposure, *HcTOE3*-OE lines displayed substantially elevated POD and SOD activities along with enhanced proline accumulation compared to WT, whereas HcmiR172e-OE lines exhibited significantly compromised antioxidant defenses and diminished proline contents. To mechanistically underpin these physiological phenotypes, qRT-PCR analysis of representative genes governing osmoregulation (*AtP5CS*), ROS detoxification (*AtPOD*, *AtCAT*), ion homeostasis and general stress tolerance (*AtSOS3*, *AtNHX1*, *AtDREB2A*, *AtRD29A*), as well as ABA-related genes (*AtRAB18*, *AtABA1*) were conducted. Notably, *HcTOE3*-OE lines showed pronounced transcriptional upregulation across this entire stress-responsive gene network relative to WT, while HcmiR172e-OE lines presented converse expression patterns with significant downregulation of these protective genes (Figure 4m–u). This antagonistic transcriptional response aligned with the stress-triggered suppression of HcmiR172e.

### 2.5. HcmiR172e-Mediated Reduction of Antioxidant and Osmotic Regulation in Arabidopsis Under Low-Temperature Treatment

To investigate the role of the HcmiR172e/*HcTOE3* module in low-temperature stress responses, phenotypic, physiological and molecular changes in HcmiR172e-OE lines were examined and compared with those of the previously reported function of its target gene *HcTOE3* [26].

**Figure 5:**
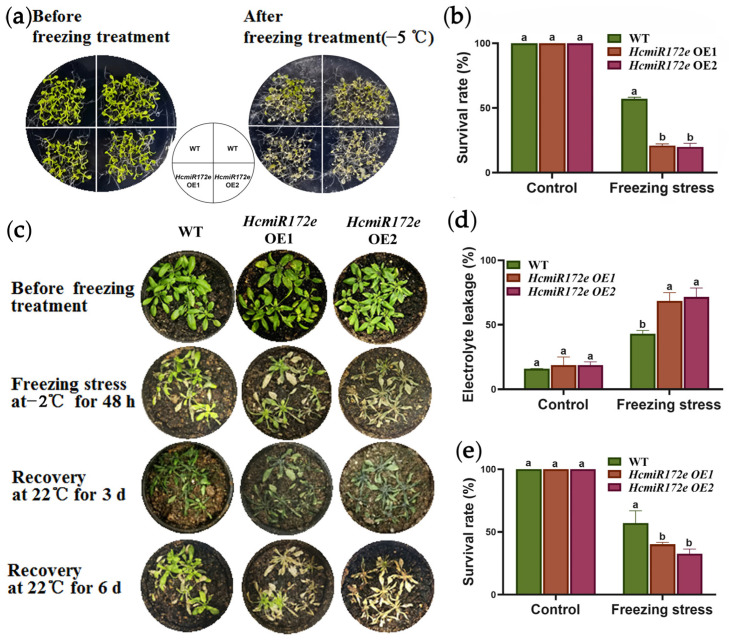
HcmiR172e overexpression compromise freezing tolerance in *Arabidopsis*. (**a**) Phenotypic comparison of WT and HcmiR172e-OE *Arabidopsis* seedlings before and after freezing treatment at −5 °C. (**b**) Survival rates of WT and HcmiR172e-OE *Arabidopsis* seedlings under control and −5 °C freezing stress. (**c**) Phenotypic changes of soil-grown plants after freezing stress (−2 °C for 48 h), and after 3 d and 6 d of recovery at 22 °C, separately. (**d**) Electrolyte leakage in WT and HcmiR172e-OE lines under control and freezing stress. (**e**) Survival rates of soil-grown plants after freezing stress. Data are shown as means ± SD (n = 3). Different letters above bars indicate significant differences among transgenic lines under low temperature treatments (*p* < 0.05, one-way ANOVA).

Freezing tolerance assays revealed that HcmiR172e-OE lines exhibited markedly reduced survival rates (25%) relative to WT (60%; Figure 5a–e), accompanied by severe wilting and necrosis. In contrast, *HcTOE3*-OE lines displayed enhanced low temperature tolerance and higher survival rates than WT. Consistently, electrolyte leakage was significantly elevated (70%; Figure 5d) and survival rates reduced (30%; Figure 5e) in HcmiR172e-OE lines under freezing stress.

**Figure 6:**
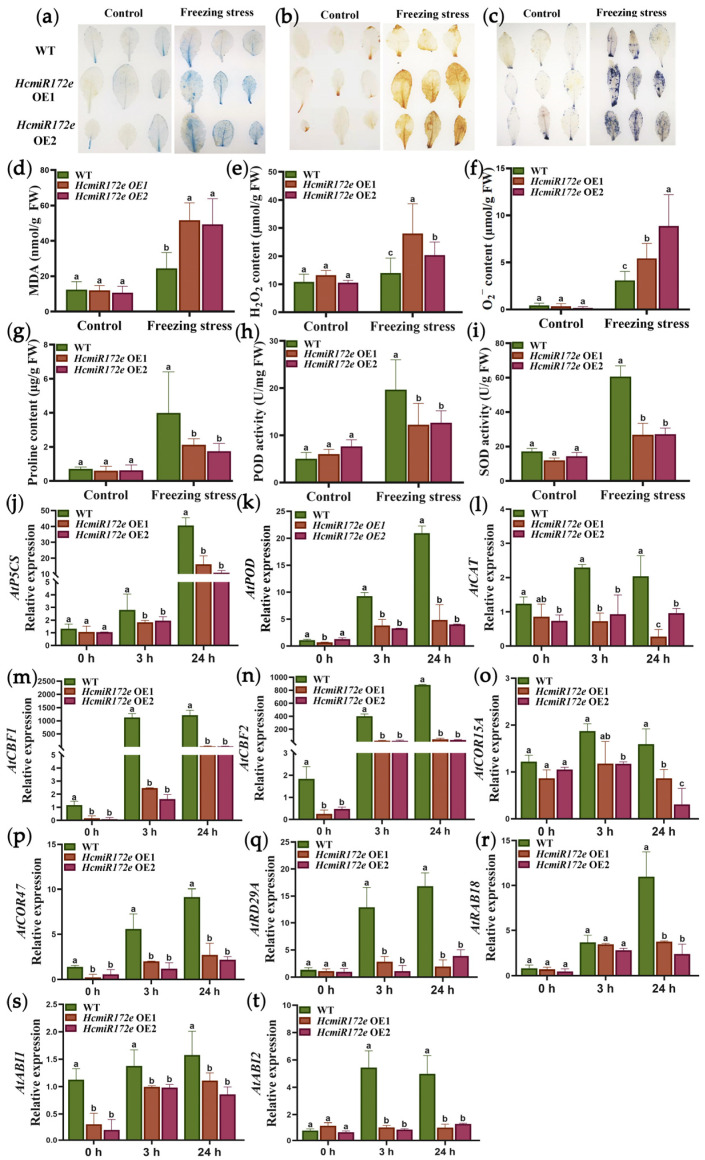
HcmiR172e-mediated repression of antioxidant and low-temperature responsive gene expression in freezing-stressed *Arabidopsis*. (**a**–**c**) Histochemical staining of 8-day-old *Arabidopsis* seedlings: (**a**) Evans blue staining for cell death, (**b**) DAB staining for H_2_O_2_ and (**c**) NBT staining for O^2•−^ in WT and HcmiR172e-OE lines under control and freezing stress conditions. (**d**–**f**) Quantification of (**d**) MDA, (**e**) H_2_O_2_ and (**f**) O^2•−^ contents. (**g**–**i**) Physiological parameters: (**g**) Proline content, (**h**) POD activity and (**i**) SOD activity. (**j**–**l**) Relative expression of antioxidant and osmotic regulation genes via qRT-PCR: (**j**) *AtP5CS*, (**k**) *AtPOD* and (**l**) *AtCAT*. (**m**–**q**) Relative expression of low-temperature responsive genes: (**m**) *AtCBF1*, (**n**) *AtCBF2*, (**o**) *AtCOR15A*, (**p**) *AtCOR47* and (**q**) *AtRD29A*. (**r**–**t**) Relative expression of ABA signaling genes: (**r**) *AtRAB18*, (**s**) *AtABI1* and (**t**) *AtABI2*. Different letters above bars indicate significant differences among transgenic lines under low temperature treatments and at different time points (*p* < 0.05, one-way ANOVA).

Meanwhile, ROS accumulation, antioxidant enzyme activities, osmotic adjustment substances and candidate gene expression were analyzed in transgenic HcmiR172e seedlings under low temperature stress. Histochemical staining with DAB, NBT and Evans blue revealed elevated accumulation of H_2_O_2_, O^2•−^ and dead cells in HcmiR172e-OE lines compared to WT (Figure 6a–c), which was further substantiated by quantitative measurements of H_2_O_2_, O^2•−^ and MDA levels (Figure 6d–f). In contrast, *HcTOE3* overexpression was reported to attenuate ROS accumulation under freezing stress [26]. Regarding antioxidant and osmotic regulation, HcmiR172e-OE lines exhibited reduced POD and SOD activities, as well as lower proline content, under freezing stress compared to WT (Figure 6g–i). This trend contrasted with *HcTOE3*-OE lines, which displayed enhanced enzyme activities and proline accumulation under low temperature stress. qRT-PCR analysis further revealed that the downregulated expression of antioxidant genes (*AtPOD*, *AtCAT*) and an osmotic regulation gene (*AtP5CS*) in HcmiR172e-OE lines under freezing stress (Figure 6j–l).

The expression of low-temperature response genes (*AtCBF1*, *AtCBF2*, *AtCOR15A*, *AtCOR47*, *AtRD29A*) and ABA signaling genes (*AtABI1*, *AtABI2*, *AtRAB18*) showed no significant differences detected between transgenic HcmiR172e *Arabidopsis* and WT under non-stress conditions. However, low temperature stress-induced expression of these genes was significantly attenuated in HcmiR172e-OE lines compared to WT (Figure 6m–t). In contrast, *HcTOE3*-OE lines exhibited upregulated expression of these genes under low temperature stress [26]. This antagonistic expression pattern observed in the HcmiR172e-OE and *HcTOE3*-OE lines in response to low temperature further confirmed that downregulation of HcmiR172e relieved the repression on *HcTOE3*, thereby positively regulated low temperature tolerance in *Arabidopsis*.

### 2.6. Role of the HcmiR172e/HcTOE3 Module in ABA Signaling Regulation

**Figure 7:**
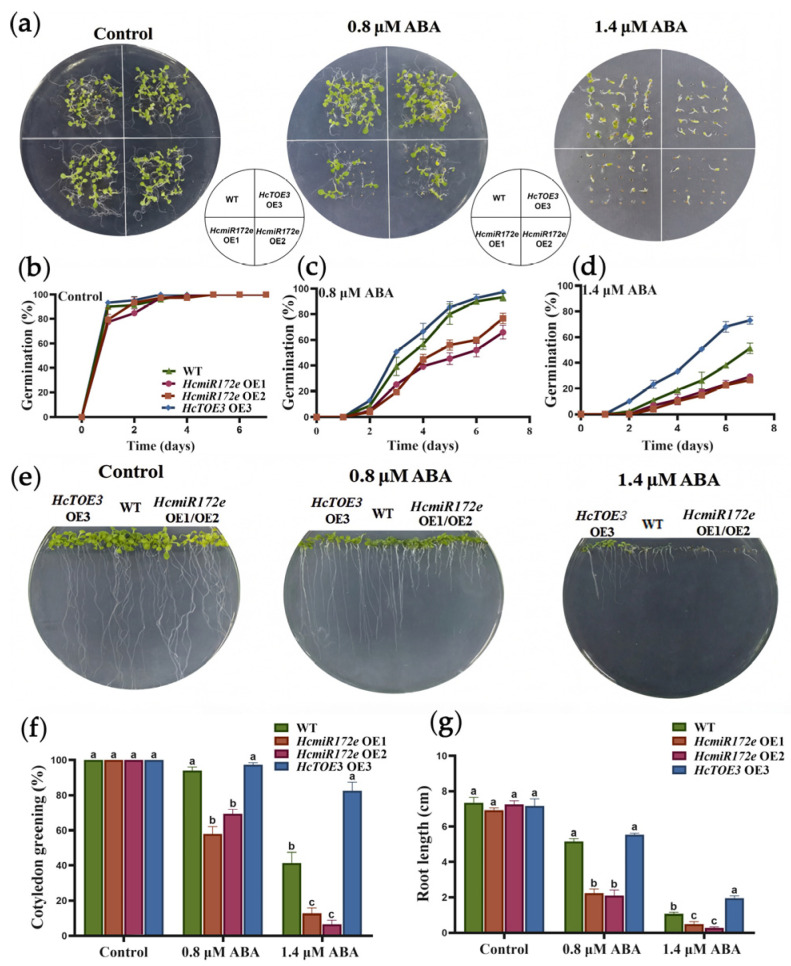
The HcmiR172e/*HcTOE3* module regulates ABA response in *Arabidopsis* seed germination and early seedling growth. (**a**–**d**) Seed germination of WT, HcmiR172e-OE and *HcTOE3*-OE *Arabidopsis* under control, 0.8 μM ABA and 1.4 μM ABA conditions: (**a**) phenotypes, (**b**) germination kinetics under control, (**c**) 0.8 μM ABA, (**d**) 1.4 μM ABA. (**e**–**g**) Post-germination growth phenotypes: (**e**) phenotypes, (**f**) cotyledon greening, (**g**) root length. Data are shown as means ± SD (n = 3). Different letters above bars indicate significant differences among transgenic lines under ABA treatment (*p* < 0.05, one-way ANOVA).

Plants rapidly accumulate endogenous ABA in response to abiotic stresses, serving as a core hormone that initiates downstream defense responses [31]. To assess the role of the HcmiR172e/*HcTOE3* module in the ABA signaling, seed germination and seedling growth under exogenous ABA treatment were examined, as *HcTOE3*-OE lines were previously shown to be insensitive to ABA [26]. Under unstressed conditions, no significant differences in germination rate, cotyledon greening rate, or root length were observed among WT, HcmiR172e-OE lines and *HcTOE3*-OE lines (Figure 7a,b).

Upon 0.8 μM and 1.4 μM ABA treatment, *HcTOE3*-OE lines consistently outperformed WT, with higher germination (96% and 70% vs. 89% and 47%), cotyledon greening (95% and 81% vs. 90% and 48%), and radicle length (5 cm and 1.8 cm vs. 4.6 cm and 1 cm) at both concentrations. In contrast, HcmiR172e-OE lines exhibited heightened ABA sensitivity, showing reduced germination (65% and 25%), cotyledon greening (65% and 7%) and radicle elongation (2.5 cm and 0.5 cm) compared to WT. WT plants displayed an intermediate phenotype (Figure 7a–g). These results demonstrated that the HcmiR172e/*HcTOE3* module was involved in ABA signaling.

## 3. Discussion

The present study identified that HcmiR172e was downregulated under various abiotic stress (Figure 1). Overexpression of HcmiR172e had no effect on seed germination and seedling growth in *Arabidopsis* but inhibited plant growth under salt, drought and freezing stresses, an effect contrary to that of *HcTOE3* overexpression. Accordingly, HcmiR172e directly cleaved *HcTOE3*; its overexpression thus enhanced this cleavage, leading to increased ROS accumulation, compromised membrane stability, and reduced synthesis of stress-responsive genes. In summary, these findings establish the HcmiR172e/*HcTOE3* module as a key regulator of plant tolerance to multiple abiotic stresses.

### 3.1. HcmiR172e Targets HcTOE3 and Negatively Regulates Stress Tolerance

The earliest evidence that miR172 cleaved AP2 subfamily was obtained in *Arabidopsis*, where five miR172 family members were identified. Each of these responded to flowering time, temperature and photoperiod [13,16,29]. AtmiR172s regulated *Arabidopsis* inflorescence meristem size by cleaving on AP2 [32]. In tomato [17], and soybean [33] miR172 was induced by abiotic stresses and enhanced drought tolerance by repressing AP2-type targets. In Rice and wheat, miR172 improved salt tolerance by inhibiting AP2 subfamily *IDS1*, via activating ROS scavenging pathway [18].

In this study, we validated the direct targeting relationship between HcmiR172e and its target gene *HcTOE3*, a member of the AP2 subfamily from *H. caspica* and confirmed the response of HcmiR172e/*HcTOE3* module to salt, drought and freezing stress in *Arabidopsis*. The significant downregulation of endogenous *AtTOE1/2/3* genes in HcmiR172e-OE lines verified the conserved targeting of miR172e to TOE family genes in *Arabidopsis*, further supporting the evolutionary conservation of this module’s targeting specificity (Figure 2 and Figure 3).

Phenotypic assays under salt and drought stresses revealed the functional antagonism between HcmiR172e and *HcTOE3*. *HcTOE3*-OE lines exhibited significantly higher germination rates, cotyledon greening rates and radicle lengths than WT under stresses conditions, while HcmiR172e-OE lines showed the opposite trend. HcmiR172e was downregulated under various abiotic-stresses, thus alleviating the HcmiR172e-mediated repression of *HcTOE3* to modulate stress tolerance. This regulatory pattern was consistent with miR172’s role as a negative regulator in stress responses in a subset of some species, such as peony (under chilling stress) and tobacco (upon viral infection) [23,34,35].

Our previous work demonstrated that *HcTOE3*-OE lines enhance freezing tolerance in *Arabidopsis* [26]. In this study, we showed that *HcTOE3* acts as a pleiotropic stress-responsive regulator conferring tolerance to salt, drought and ABA responses.

### 3.2. Antagonistic Regulation of the HcmiR172e/HcTOE3 Module in Physiological and Molecular Responses to Salt, Drought and Freezing Stresses

Under salt, drought and freezing stress, plants inherently accumulated ROS that triggered lipid peroxidation and cellular damage [36,37]. In this study, HcmiR172e and *HcTOE3* exhibited diametrically opposed effects on ROS detoxification: *HcTOE3* significantly enhanced the activity of antioxidant enzymes (SOD, POD) and upregulated the transcription of genes (*AtPOD*, *AtCAT*), thereby reducing ROS and MDA accumulation (Figure 4 and Figure 6) in transgenic *Arabidopsis* [26]. In contrast, HcmiR172e-OE lines showed impaired ROS detoxification and elevated ROS/MDA levels, which exacerbated oxidative damage. This regulatory pattern displayed species-specific divergence in the miR172/*TOE* module. In rice, OsmiR172 enhances salt tolerance by inhibiting *OsIDS1* to activate ROS scavenging [18]. While in tree peony, tobacco and lily overexpression of miR172 reduced the activities of POD, SOD and CAT, and proline content and increased the concentration of O^2•−^ [23,24,34], this phenomenon was consistent with that observed for HcmiR172e-OE lines.

Under salt and drought stress, osmotic adjustment, particularly through ion and solute balance, constitutes another critical pathway for stress tolerance [38]. In our study, *HcTOE3*-OE lines mitigated stresses by promoting proline biosynthesis which enhanced proline content (Figure 4l). HcmiR172e overexpression acted oppositely. However, the regulatory pattern varied in a species-specific manner. In wheat and rice, miR172-overexpression enhanced osmotic adjustment by accumulating proline content [18,33]. *HcTOE3*-OE lines also upregulated key salt-responsive genes: *AtSOS3* (a core component of the SOS pathway that mediates Na^+^ exclusion) [39] and *AtNHX1* (a vacuolar Na^+^/H^+^ antiporter that sequesters Na^+^) [40]. In contrast, HcmiR172e-OE lines showed significantly reduced expression of these genes, leading to impaired salt tolerance. *HcTOE3*-OE lines induced the expression of *AtDREB2A* (a transcription factor activating drought-responsive genes) [41] and *AtRD29A* (a marker gene responsive to drought stress) [42], while HcmiR172e-OE lines repressed the expression of these genes, resulting in increased water loss and drought sensitivity.

Electrical conductivity and cell membrane permeability are key indicators for plant freezing stress [43]. The C-repeat binding factor (CBF) and Cold-Regulated Gene (COR) families were key gene families involved in cold stress response [44,45]. Under freezing stress, the HcmiR172e/*HcTOE3* module integrated the CBF-COR pathway, that was *HcTOE3*-OE lines upregulated *AtCBF1/2* [46] and their downstream targets *AtCOR15A/47* (cryoprotective proteins) (Figure 6) [47,48]. HcmiR172e-OE lines, however, exhibited attenuated induction of these genes (*AtCBF1*, *AtCBF2*, *AtCOR15A*, *AtCOR47*) [26,49].

*HcTOE3* activated downstream functional genes (*AtRD29A*, *AtRAB18*, *AtABA1*), which enables the miR172e/*TOE3* module to respond to salt, drought and freezing stress through both ABA-dependent and ABA-independent pathways [50]. The ABA sensitivity assay further confirmed the module’s involvement in ABA-dependent signaling. Under exogenous ABA treatment, HcmiR172e-OE lines showed concentration-dependent reductions in germination rate, cotyledon greening rate and root length, while *HcTOE3*-OE lines maintained stable growth performance comparable to unstressed WT. However, HcmiR172-OE lines exhibited upregulated expression of *AtRD29A*, *AtRAB18*, *AtCOR15A* and other genes under salt and drought stresses, which was completely opposite to the response of HcmiR172e to ABA [33].

### 3.3. Regulatory Mechanism of the HcmiR172e/HcTOE3 Module

The antagonistic regulation of HcmiR172e and *HcTOE3* likely reflects a finely tuned growth-stress trade-off. Under normal growth conditions, HcmiR172e represses *HcTOE3* to prioritize reproductive growth, while under stress, its downregulation releases *HcTOE3* to promote stress tolerance [26]. This dynamic balance was reported in the miR172/*AP2* module across plant species [30,34,35,51]. While we have verified that HcmiR172e regulates plant physiological processes and downstream gene expression by targeting *HcTOE3*, the specific direct target genes regulated by *HcTOE3* as a transcription factor remain to be further investigated.

**Figure 8:**
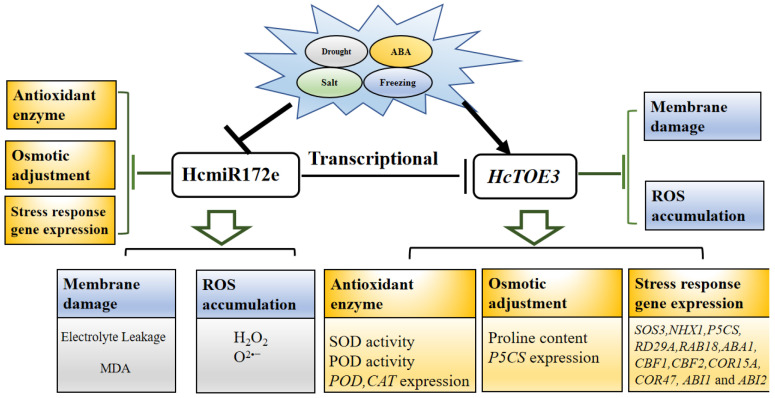
Regulation of the HcmiR172e/*HcTOE3* module in *Arabidopsis* salt, drought, freezing and ABA treatments. *HcmiR172e* negatively regulates stress tolerance by repressing *HcTOE3*, leading to membrane damage (elevated electrolyte leakage and MDA content), ROS accumulation (H_2_O_2_, O^2•−^), impaired antioxidant enzyme activity (SOD, POD), osmotic adjustment, and stress-responsive gene expression (*SOS3*, *NHX1*, *RD29A* etc.). In contrast, *HcTOE3* promotes stress tolerance by enhancing antioxidant defense, osmotic balance, and stress-responsive gene induction, thereby reducing ROS accumulation and membrane damage.

Integrating the above findings, we propose that the HcmiR172e/HcTOE3 module participates in ROS scavenging, osmotic adjustment and in ABA signaling to maintain cellular homeostasis, thereby expanding our understanding of how halophytes coordinate multi-stress responses (Figure 8).

## 4. Materials and Methods

### 4.1. Stress Treatment and Gene Expression Detection in Assimilating Branches of H. caspica

*H. caspica* seeds were collected from the semi-desert area with extreme saline-alkali conditions at the edge of the Gurbantünggüt Desert, Xinjiang, China. Healthy seeds were sown in pots containing a substrate mixture (perlite: vermiculite: flower soil = 1:1:3) and cultivated under natural light (25–28 °C). Eight-week-old *H. caspica* were exposed to 4 °C chilling injury, 0 °C freezing point, −2 °C freezing stress, 45 °C high temperature, 600 mM NaCl, 1000 mM mannitol, 100 μM methyl viologen (MV) and 300 μM ABA for 0 h, 3 h and 24 h [52]. Assimilating branches were harvested, frozen immediately in liquid nitrogen, and stored for subsequent total RNA extraction and qRT-PCR analysis [53].

### 4.2. Cloning and Characterization of HcmiR172e and Its Precursor, and Prediction of Target Gene HcTOE3

Based on the pre-miR172e sequences from different species in the miRBase database, including *Arabidopsis* and ten other plant species, precursor primers (miR172e-F and miR172e-R) for homologous cloning were designed (Supplementary Table S1). Subsequently, 5′-RACE primers (miR172e-5′RACE-GSP and miR172e-5′RACE-NGSP) were designed based on the sequence obtained from homologous cloning. These primers were used to acquire the 5′end sequence of the HcmiR172e precursor. The full-length gene sequence was then obtained by splicing the homologous cloning fragment of the HcmiR172e precursor with the 5′-RACE sequence. The amplified product was ligated into the pMD-19T vector for sequencing and verification. Using the psRNATarget online tool (https://www.zhaolab.org/psRNATarget/, accessed on 1 December 2025) and *H. caspica* transcriptome data, *HcTOE3* was predicted as the target gene of HcmiR172e.

The *HcTOE3*-OE lines used in this study were generated in our laboratory previously [26]. For the construction of HcmiR172e-OE lines, the precursor region of HcmiR172e was cloned into the pCAMBIA2300 vector and transformed into *Arabidopsis* (Col-0) via the floral dip method. Subsequently, transgenic seeds of the T3 generation that were homozygous, single-copy and highly expression were screened for subsequent experiments [53].

### 4.3. Tobacco Transient Expression Assay for Validating HcmiR172e-HcTOE3 Interaction

To confirm the direct cleavage of *HcTOE3* by HcmiR172e, a tobacco transient expression assay was performed. The constructs pCAMBIA2300-pre-HcmiR172e and pBI121-*HcTOE3* were first introduced into *Agrobacterium tumefaciens* strain EHA105. The bacterial strains were cultured in YEB medium until the OD_600_ reached 0.8–1.0, after which the cells were harvested by centrifugation and resuspended in MS buffer to a final OD_600_ of 0.5. Subsequently, equal volumes of the suspensions containing EHA105-pCAMBIA2300-pre-HcmiR172e (OD_600_ 1.0) and EHA105-pBI121-*HcTOE3* (OD_600_ 1.0) were mixed. A 500 μL aliquot of the mixed agrobacterial suspension was then infiltrated into the leaves of four-week-old *Nicotiana benthamiana* plants. The infiltrated plants were incubated in the dark for 2 days, followed by normal growth conditions for 3 days before GUS expression analysis. For GUS staining, the infiltrated leaf samples were immersed in GUS staining solution and incubated overnight at 37 °C with shaking at 100 rpm. Finally, the chlorophyll was removed by ethanol soaking to allow for clear observation of the staining results [54].

### 4.4. 5′RLM-RACE Technology to Determine the Cleavage Target Site of HcmiR172e on HcTOE3

The method used in this study was improved based on the 5′ RLM-RACE technique [55] (Figure 2). T4 RNA ligase (Ambion, Burlington, ON, Canada) was used to add an adapter to the 3′ hydroxyl end of total RNA isolated from *H. caspica* assimilating branches. A specific primer complementary to the adapter were used for reverse transcription to obtain cDNA with an adapter sequence. Nested PCR amplification was then performed twice with adapter-specific and *HcTOE3*-specific primers using this cDNA as a template. The second round amplification products were ligated into the pMD-19T vector for sequencing, and the sequencing results were analyzed to determine the cleavage target site of HcmiR172e on *HcTOE3* (Figure 2). All primers used in this study was listed in the Supplementary Table S1.

### 4.5. qRT-PCR Detection of Gene Expression Levels

Total RNA from plant samples was extracted using the RNA prep pure Plant Kit (TianGen, Beijing, China). The stem-loop method was used for reverse transcription of miRNA172e, with *HcU6* and *Hc5SRNA* as the internal reference genes for *H. caspica*. Other genes were reverse-transcribed using the One-Step gDNA Removal and cDNA Synthesis SuperMix (TianGen, Beijing, China) [56], with *HcUBQ10* and *AtActin* as internal controls. qRT-PCR was performed on the CFX96 Touch Real-Time PCR System (Bio-Rad, Hercules, CA, USA) using PerfectStart Green qPCR SuperMix (Transgen, BeiJing, China), with three biological replicates for each sample. The 2^−ΔΔCT^ method was used to calculate the relative expression levels of the genes [57].

### 4.6. Treatment of Transgenic Arabidopsis

*Arabidopsis* seeds were surface-sterilized with 75% ethanol for 30 s, rinsed with sterile water three times, then treated with 10% sodium hypochlorite for 5 min with gentle shaking, and finally rinsed with sterile water five times. Seeds were sown on 1/2 MS medium and stratified at 4 °C for 3 days and then moved to a greenhouse at 22 °C with a light/dark cycle of 16 h/8 h. After two weeks of growth, the plants were transferred to pots for cultivation with a substrate mixture. After 8 days of vertical cultivation, seedling root length was measured.

For germination and growth assays under stress conditions, seeds were sown on MS medium supplemented with NaCl (150 mM), mannitol (350 mM) and ABA (0.8 μM, 1.4 μM). Each treatment comprised 30 seeds per replicate, with three biological replicates. Daily germination rates were recorded, and the greening rate of cotyledons was counted after 7 days, while related physiological and biochemical indicators were measured and qRT-PCR detection was performed after the same treatment period.

For freezing tolerance assays, *Arabidopsis* plants were potted in the same weight of substrate as the pots, and the plants were placed in a −2 °C environment for 48 h and then recovered at 22 °C for 3 and 6 days. Photos were taken, and the survival rate of the plants was statistically analyzed (three independent experiments, each with 40 seedlings per replicate). Physiological indicators were measured, and leaf samples were collected for qRT-PCR detection.

### 4.7. Physiological and Biochemical Indicator Measurements

*Arabidopsis* leaves were immersed in 1 mg/mL DAB, 0.5 mg/mL NBT and 0.25% Evans Blue solutions, respectively, and stained for 2 h at 37 °C in the dark. The leaves were placed in anhydrous ethanol at boiling temperature for 30 min to remove chlorophyll. Ten leaves were used for each treatment.

Fresh *Arabidopsis* leaves (0.1 g) were cut into small pieces and soaked in 50 mL of ultra-pure water for 24 h, and the solution conductivity (C1) was measured using a conductivity meter DSS-307 (Leici, Shanghai, China). After boiling for 1 h, the solution conductivity (C2) was measured again, and the ion leakage rate was calculated as C1/C2 × 100%.

The content of chlorophyll, MDA, H_2_O_2_, O^2•−^ and proline was measured, and the enzyme activities of POD, SOD and CAT were determined according to the manufacturer’s instructions (Solarbio, Beijing, China). Each treatment had three biological replicates, and 0.1 g of *Arabidopsis* leaves were collected for each biological replicate.

### 4.8. Statistical Analysis

Data analysis was performed using SPSS Statistics 20 (IBM, Armonk, NY, USA). For the relative expression levels of miR172e in *H. caspica*, one-way ANOVA was conducted to compare the 0 h control group with each treatment time point (3 h, 24 h). Prior to ANOVA, the Shapiro–Wilk test was used to verify the normality of the data, and Levene’s test was performed to assess the homogeneity of variances. Duncan’s test was employed for multiple comparisons. Differences were considered statistically significant at *p* < 0.05. All experiments were performed with at least three biological replicates.

## 5. Conclusions

In this study, a key HcmiR172e/*HcTOE3* regulatory module was identified in the halophyte *H. caspica*, revealing a novel mechanism by which *HcTOE3* confers multiple abiotic stress tolerance (salt, drought and freezing) in plants through antagonistic regulation. HcmiR172e directly cleaves the *HcTOE3* transcript. Under various stresses, the expression of HcmiR172e was significantly downregulated, releasing the inhibition of *HcTOE3*. Functional verification showed that overexpression of *HcTOE3* significantly improved stress tolerance in *Arabidopsis thaliana* by enhancing reactive oxygen species scavenging, osmotic adjustment, and the expression of stress-responsive genes, whereas overexpression of HcmiR172e had the opposite effect. This module maintains cellular homeostasis by participating in ABA signaling pathways. Its regulatory mode reflects the unique stress adaptation strategy of halophytes, providing potential targets for the genetic improvement of crop stress resistance.

## Data Availability

The datasets used in the current study are available from the corresponding author on reasonable request.

## Supplementary Materials

The following supporting information can be downloaded at: https://www.mdpi.com/article/10.3390/plants15071087/s1, Supplementary Table S1: Primers used in this study.

## Abbreviations

The following abbreviations are used in this manuscript:

POD	Peroxidase
SOD	Superoxide Dismutase
CAT	Catalase
SD	Standard Deviation
ANOVA	Analyzed Using One-way Analysis of Variance

## References

[1] Zhang H.M., Zhu J.H., Gong Z.Z., Zhu J.K. (2021). Abiotic stress responses in plants. Nat. Rev. Genet..

[2] Wang D.J., Gao Y., Sun S.M., Lu X., Li Q.S., Li L.W., Wang K., Liu J.H. (2022). Effects of salt stress on the antioxidant activity and malondialdehyde, solution protein, proline, and chlorophyll contents of three species. Life.

[3] Considine M.J., Foyer C.H. (2021). Stress effects on the reactive oxygen species-dependent regulation of plant growth and development. J. Exp. Bot..

[4] Wang Z.L., Wu D., Hui M., Wang Y., Han X., Yao F., Cao X., Li Y.H., Li H., Wang H. (2022). Screening of cold hardiness-related indexes and establishment of a comprehensive evaluation method for grapevines (*V. vinifera*). Front. Plant Sci..

[5] Liu J.k., Shen F.B., Jin Q.W., Deng H., Wang F., Muhammad Umer Bin Muhammad I., Huang H.T., Gao Y.F. (2026). Overexpression of *SlANT1* enhances drought tolerance in tomato through anthocyanin-mediated antioxidant protection and metabolic reprogramming. Plants.

[6] Cao W.X., Yang L.M., Zhuang M., Lv H.H., Wang Y., Zhang Y.Y., Ji J.L. (2024). Plant non-coding RNAs: The new frontier for the regulation of plant development and adaptation to stress. Plant Physiol. Biochem..

[7] Shriram V., Kumar V., Devarumath R.M., Khare T.S., Wani S.H. (2016). MicroRNAs as potential targets for abiotic stress tolerance in plants. Front. Plant Sci..

[8] Bartel D.P. (2004). MicroRNAs: Genomics, biogenesis, mechanism, and function. Cell.

[9] Zhao Y.X., Kuang Z., Wang Y., Li L., Yang X.Z. (2021). MicroRNA annotation in plants: Current status and challenges. Brief. Bioinform..

[10] Yang Z., Dong T., Dai X., Wei Y., Fang Y., Zhang L., Zhu M., Nawaz G., Cao Q., Xu T. (2022). Comparative analysis of salt responsive microRNAs in two sweetpotato [*Ipomoea batatas* (L.) Lam.] cultivars with different salt stress resistance. Front. Plant Sci..

[11] Niu M.X., Feng C.H., He F., Zhang H., Bao Y., Liu S.J., Liu X., Su Y.Y., Liu C., Wang H.L. (2024). The miR6445-*NAC029* module regulates drought tolerance by regulating the expression of glutathione S-transferase U23 and reactive oxygen species scavenging in Populus. New Phytol..

[12] Thiebaut F., Rojas C.A., Almeida K.L., Grativol C., Domiciano G.C., Lamb C.R.C., de Almeida Engler J., Hemerly A.S., Ferreira P.C.G. (2012). Regulation of miR319 during cold stress in sugarcane. Plant Cell Environ..

[13] Chen X.M., Lian H., Wang L., Ma N., Zhou C.M., Han L., Zhang T.Q., Wang J.W. (2021). Redundant and specific roles of individual miR172 genes in plant development. PLoS Biol..

[14] Han Y., Wang L., Zhang X.L., Korpelainen H., Li C.Y. (2013). Sexual differences in photosynthetic activity, ultrastructure and phytoremediation potential of *Populus cathayana* exposed to lead and drought. Tree Physiol..

[15] Lauter N., Kampani A., Carlson S., Goebel M., Moose S.P. (2005). *MicroRNA172* down-regulates *glossy15* to promote vegetative phase change in maize. Proc. Natl. Acad. Sci. USA.

[16] Nowak K., Moronczyk J., Grzyb M., Szczygiel-Sommer A., Gaj M.D. (2022). miR172 regulates *WUS* during somatic embryogenesis in *Arabidopsis* via AP2. Cells.

[17] Bansal C., Kumar A., Shrivastava M., Mathur S. (2024). Functional diversification of miR172 isoforms in tomato under abiotic stress. Environ. Exp. Bot..

[18] Cheng X.L., He Q., Tang S., Wang H.R., Zhang X.X., Lv M.J., Liu H.F., Gao Q., Zhou Y., Wang Q. (2021). The miR172/*IDS1* signaling module confers salt tolerance through maintaining ROS homeostasis in cereal crops. New Phytol..

[19] Yang R.R., Zeng Y.L., Yi X.Y., Zhao L.J., Zhang Y.F. (2015). Small RNA deep sequencing reveals the important role of microRNAs in the halophyte *Halostachys caspica*. Plant Biotechnol. J..

[20] Ma N., Sun P., Li Z.Y., Zhang F.J., Wang X.F., You C.X., Zhang C.L., Zhang Z.L. (2024). Plant disease resistance outputs regulated by AP2/ERF transcription factor family. Stress Biol..

[21] Feng K., Hou X.L., Xing G.M., Liu J.X., Duan A.Q., Xu Z.S., Li M.Y., Zhuang J., Xiong A.S. (2020). Advances in AP2/ERF super family transcription factors in plant. Crit. Rev. Biotechnol..

[22] Cao S.L., Wang Y., Li X.T., Gao F., Feng J.C., Zhou Y.J. (2020). Characterization of the AP2/ERF transcription factor family and expression profiling of DREB subfamily under cold and osmotic stresses in *Ammopiptanthus nanus*. Plants.

[23] Jiao B.L., Peng Q.D., Wu B.J., Liu S.C., Zhou J.Y., Yuan B.W., Lin H.H., Xi D.H. (2024). The miR172/*TOE3* module regulates resistance to tobacco mosaic virus in tobacco. Plant J..

[24] Li B.X., Liu Y.N., Liang H.Y., Wang X.H., Wang S.T., Shen J.J., Hou X.G., Guo L.L. (2025). PomiR172d-*PoARR* module regulates the drought response through the reactive oxygen pathway in tree peony. Hortic. Res..

[25] Han Z.X., Li L., Xu X.W., Wei X. (2013). Adaptation of *Halostachys caspica* to different soil salt and water contents in salted desert (China). J. Northwest A&F Univ. (Nat. Sci. Edn.).

[26] Yin F.L., Zeng Y.L., Ji J.Y., Wang P.J., Zhang Y.F., Li W.H. (2021). The halophyte *Halostachys caspica* AP2/ERF transcription factor *HcTOE3* positively regulates freezing tolerance in *Arabidopsis*. Front. Plant Sci..

[27] Lambert N.J., Gu S.G., Zahler A.M. (2011). The conformation of microRNA seed regions in native microRNAs is prearranged for presentation to mRNA targets. Nucleic Acids Res..

[28] Bartel D.P. (2009). MicroRNAs: Target recognition and regulatory functions. Cell.

[29] O’Maoiléidigh D.S., van Driel A.D., Singh A., Sang Q., Le Bec N., Vincent C., de Olalla E.B.G., Vayssières A., Branchat M.R., Severing E. (2021). Systematic analyses of the miR172 family members of *Arabidopsis* define their distinct roles in regulation of APETALA2 during floral transition. PLoS Biol..

[30] Mittler R., Zandalinas S.I., Fichman Y., Van Breusegem F. (2022). Reactive oxygen species signalling in plant stress responses. Nat. Rev. Mol. Cell Biol..

[31] Shintani M., Tamura K., Bono H. (2024). Meta-analysis of public RNA sequencing data of abscisic acid-related abiotic stresses in *Arabidopsis thaliana*. Front. Plant Sci..

[32] Sang Q., Vayssières A., O’Maoiléidigh D.S., Yang X., Vincent C., de Olalla E.B.G., Cerise M., Franzen R., Coupland G. (2022). MicroRNA172 controls inflorescence meristem size through regulation of APETALA2 in *Arabidopsis*. New Phytol..

[33] Li W.B., Wang T., Zhang Y.H., Li Y.G. (2016). Overexpression of soybean miR172c confers tolerance to water deficit and salt stress, but increases ABA sensitivity in transgenic *Arabidopsis thaliana*. J. Exp. Bot..

[34] Wang X.S., Li Q., Zhu H.R., Song M.Q., Zhang K.Z., Ge W. (2023). Molecular mechanisms of miR172a and its target gene *LbrTOE3* regulating maturation in *Lilium*. Planta.

[35] Zhang Y.X., Gao L.Q., Wang Y.Y., Niu D.M., Yuan Y.C., Liu C.Y., Zhan X.M., Gai S.P. (2023). Dual functions of PsmiR172b-*PsTOE3* module in dormancy release and flowering in tree peony (*Paeonia suffruticosa*). Hortic. Res..

[36] Bai X.F., Dai L.Q., Sun H.M., Chen M.T., Sun Y.L. (2019). Effects of moderate soil salinity on osmotic adjustment and energy strategy in soybean under drought stress. Plant Physiol. Biochem..

[37] Li J.X., Bai X.M., Ran F., Zhang C.Z., Yan Y.B., Li P., Chen H. (2024). Effects of combined extreme cold and drought stress on growth, photosynthesis, and physiological characteristics of cool-season grasses. Sci. Rep..

[38] Munns R., Passioura J.B., Colmer T.D., Byrt C.S. (2020). Osmotic adjustment and energy limitations to plant growth in saline soil. New Phytol..

[39] Yang D.H., Song L.Y., Hu J., Yin W.B., Li Z.G., Chen Y.H., Su X.H., Wang R.C., Hu Z.M. (2012). Enhanced tolerance to NaCl and LiCl stresses by over-expressing *Caragana korshinskii* sodium/proton exchanger 1 (*CkNHX1*) and the hydrophilic C terminus is required for the activity of *CkNHX1* in *Atsos3-1* mutant and yeast. Biochem. Biophys. Res. Commun..

[40] Liu Y., Hou Q., Dong K.L., Chen Y., Wang Z.H., Xie S.D., Wu S.J., Zhang X.Q., Yu S.Z., Yang Z.X. (2024). Overexpression of *AtNHX1* increases leaf potassium content by improving enrichment capacity in tobacco (*Nicotiana tabacum*) roots. Funct. Plant Biol..

[41] Souza W.R., Oliveira N.G., Vinecky F., Ribeiro A.P., Basso M.F., Casari R., da Cunha B., Duarte K.E., Santiago T.R., Martins P.K. (2019). Field evaluation of *AtDREB2A* CA overexpressing sugarcane for drought tolerance. J. Agron. Crop Sci..

[42] Wang D., Yang Z.J., Feng M.Y., Yang W.W., Qu R., Nie S.M. (2024). The overexpression of *SlBRI1* driven by *AtRD29A* promoter-transgenic plants improves the chilling stress tolerance of tomato. Planta.

[43] Cha S.J., Park H.J., Kwon S.J., Lee J.K., Park J.H. (2021). Early detection of plant stress using the internal electrical conductivity of *Capsicum annuum* in response to temperature and salinity stress. Plant Growth Regul..

[44] Javier B., Julio S. (2017). CBFs at the crossroads of plant hormone signaling in cold stress response. Mol. Plant.

[45] Ding Y.L., Shi Y.T., Yang S.H. (2024). Regulatory networks underlying plant responses and adaptation to cold stress. Annu. Rev. Genet..

[46] Storani L., Hernando C.E., Staneloni R.J., Ploschuk E., Rugnone M.L., Striker G.G., Casal J.J., Chernomoretz A., Yanovsky M.J. (2015). *AtCBF1* overexpression confers tolerance to high light conditions at warm temperatures in potato plants. Am. J. Potato Res..

[47] Wan F.X., Pan Y., Li J.H., Chen X.F., Pan Y.L., Wang Y.Q., Tian S.B., Zhang X.G. (2014). Heterologous expression of *Arabidopsis* C-repeat binding factor 3 (*AtCBF3*) and cold-regulated 15A (*AtCOR15A*) enhanced chilling tolerance in transgenic eggplant (*Solanum melongena* L.). Plant Cell Rep..

[48] Wei J.p., Zheng G.Q., Yu X.W., Liu S.S., Dong X.Y., Cao X.D., Fang X.L., Li H., Jin J.J., Mi W.B. (2021). Comparative transcriptomics and proteomics analyses of leaves reveals a freezing stress-responsive molecular network in winter rapeseed (*Brassica rapa* L.). Front. Plant Sci..

[49] Islam W., Adnan M., Alomran M.M., Qasim M., Waheed A., Alshaharni M.O., Zeng F. (2024). Plant responses to temperature stress modulated by microRNAs. Physiol. Plant.

[50] Nonogaki H. (2010). MicroRNA gene regulation cascades during early stages of plant development. Plant Cell Physiol..

[51] Yan Z., Hossein M.S., Wang J., Valdés López O., Liang Y., Libault M., Qiu L.J., Stacey G. (2013). miR172 regulates soybean nodulation. Mol. Plant-Microbe Interact..

[52] Cao J., Maitirouzi A., Feng Y.D., Zhang H., Heng Y.Q., Zhang J.B., Wang Y. (2025). Heterologous expression of *Halostachys caspica* pathogenesis-related protein 10 increases salt and drought resistance in transgenic *Arabidopsis thaliana*. Plant Mol. Biol..

[53] Chen X.M. (2004). A microRNA as a translational repressor of APETALA2 in *Arabidopsis* flower development. Science.

[54] Zhang Y.F., Yang R.R., Zeng Y.L. (2016). Cloning, expression and correlated analysis of HcmiR172e with predicted target gene *HcTOE3* in *Halostachys caspica* under salt stress (China). Plant Physiol. J..

[55] Adamopoulos P.G., Tsiakanikas P., Stolidi I., Scorilas A. (2022). A versatile 5′ RACE-Seq methodology for the accurate identification of the 5′ termini of mRNAs. BMC Genom..

[56] Wang Y.N., Liang C.Z., Meng Z.G., Li Y.Y., Abid M.A., Askari M., Wang P., Wang Y., Sun G.Q., Cai Y.P. (2019). Leveraging *Atriplex hortensis* choline monooxygenase to improve chilling tolerance in cotton. Environ. Exp. Bot..

[57] Livak K.J., Schmittgen T.D. (2001). Analysis of relative gene expression data using real-time quantitative PCR and the 2^−ΔΔCT^ method. Methods.

