# Correction: Wang et al. A miR172e/*TOE3* Module from the Halophyte *Halostachys caspica* Regulates Plant Multiple Abiotic Stress Tolerance via Cellular Homeostasis. *Plants* 2026, *15*, 1087

**DOI:** 10.3390/plants15132007

**Published:** 2026-06-29

**Authors:** Yadi Wang, Jieyun Ji, Youling Zeng

**Affiliations:** Xinjiang Key Laboratory of Biological Resources and Genetic Engineering, College of Life Science and Technology, Xinjiang University, Urumqi 830017, China; 107556524190@stu.xju.edu.cn (Y.W.); jjy960922@outlook.com (J.J.)

In the original publication [1], there was a mistake in the legend for Figure 1 (Figure 1a and Figure 1c were swapped). The correct legend appears below: (**a**) 4 °C Chilling injury, (**b**) 0 °C Freezing point, (**c**) −2 °C Freezing stress.

Because some descriptions in the original publication were unclear, the revisions make them more precise. These changes—restated descriptions, consolidated paragraphs, and updated experimental details—appear in Abstract, Results 2.2, Results 2.5, Results 2.6, Materials and Methods 4.2, Materials and Methods 4.4, Materials and Methods 4.5, and Discussion sections. The scientific conclusions remain unaffected.

A correction has been made to the Abstract, including some grammatical corrections and the description of RLM-RACE cleavage. The updates are as follows:

Our previous work demonstrated that *HcTOE3* was strongly upregulated in the assimilating branches of *H. caspica* under salt, drought, high temperature, low temperature, oxidative stress, and abscisic acid (ABA) application.

A correction has been made to Results 2.2. The Targeted Cleavage of *HcTOE3* by HcmiR172e Through Experimental Methods. The full paragraph is updated as follows:

No staining signal was observed in leaves when expressing HcmiR172e alone, transformed with *Agrobacterium* containing the construct of pCAMBIA2300-*pre-HcmiR172e*, whereas leaves transformed with the empty vector (pCAMBIA1304-*GUS*) exhibited strong staining (Figure 2a,b), indicating that HcmiR172e mediated the cleavage and degradation of *HcTOE3*. RLM-RACE further confirmed that *HcTOE3* was precisely cleaved at the 1263 nt site within its ORF, with this cleavage site detected in seven out of eight clones (Figure 2c).

A correction has been made to Results 2.5. HcmiR172e-Mediated Reduction of Antioxidant Defense and Osmotic Regulation in *Arabidopsis* Under Low-Temperature Treatment. The gene expression in the fourth paragraph is updated as follows:

The expression of low-temperature response genes (*AtCBF1*, *AtCBF2*, *AtCOR15A*, *AtCOR47*, *AtRD29A*) and ABA signaling genes (*AtABI1*, *AtABI2*, *AtRAB18*) under freezing stress was significantly attenuated in HcmiR172e-OE plants compared to WT (Figure 6m–t), while these genes were significantly upregulated in *HcTOE3*-OE plants [25]. These data further confirmed that the HcmiR172e/*HcTOE3* module participates in the regulation of the low-temperature tolerance.

The original redundant description about gene expression under non-stress conditions has been omitted and the paragraph has been consolidated.

A correction has been made to several sentences to improve data clarity in Results 2.6. Role of the HcmiR172e/*HcTOE3* Module in ABA Signaling. The updates are as follows:

In assessing the role of the HcmiR172e/*HcTOE3* module in the ABA signaling, *HcTOE3*-OE *Arabidopsis* was insensitive to ABA, showing increased germination (96% and 70%), cotyledon greening (95% and 81%) and radicle elongation (5 cm and 1.8 cm) compared to WT under 0.8 μM and 1.4 μM ABA treatments during the stage of seed germination; however, HcmiR172e-OE *Arabidopsis* was more sensitive, with lower germination (65% and 25% vs. 89% and 47%), cotyledon greening (65% and 7% vs. 90% and 48%), and radicle length (2.5 cm and 0.5 cm vs. 4.6 cm and 1 cm) than WT at both concentrations (Figure 7).

A correction has been made to Materials and Methods 4.2. Tobacco Transient Expression Assay for Validating HcmiR172e-*HcTOE3* Interaction. The full paragraph is updated as follows:

Here, the HcmiR172e precursor was inserted into the pCAMBIA2300 vector to generate pCAMBIA2300-*pre-HcmiR172e*. The *HcTOE3* with a predicted targeting region (3′ end of ORF) was cloned into the pCAMBIA1304 vector harboring the *GUS* reporter gene, yielding the pCAMBIA1304-*HcTOE3-GUS* fusion construct. These constructs of pCAMBIA2300-*pre-HcmiR172e*, pCAMBIA1304-*HcTOE3-GUS*, and the pCAMBIA1304-*GUS* were individually introduced into *Agrobacterium tumefaciens* strain EHA105, and corresponding transformed bacteria, divided into four groups (I: pCAMBIA2300-*pre-HcmiR172e*; II: pCAMBIA1304-*GUS*; III: pCAMBIA1304-*HcTOE3-GUS*; IV: pCAMBIA2300-*pre-HcmiR172e* + pCAMBIA1304-*HcTOE3-GUS*), were cultured in YEB liquid medium to an OD_600_ of 1.0, separately, then harvested by centrifugation, and resuspended in MS buffer to a final OD_600_ of 0.5. For co-injection with two kinds of bacteria containing pCAMBIA2300-*pre-HcmiR172e* and pCAMBIA1304-*HcTOE3-GUS*, these bacteria were individually resuspended to an OD_600_ of 1.0, mixed at a 1:1 ratio, and then used for injection.

Incomplete experimental details and ambiguous descriptions in the original text have been supplemented and standardized.

A correction has been made to Materials and Methods 4.4. qRT-PCR Detection for Gene Expression. The updates are as follows:

For detection of miR172e in *H. caspica* and *Arabidopsis*, reverse transcription was performed using a stem-loop method with the primer: GTCGTATCCAGTGCAGGGTCCGAGGTATTCGCACTGGATACGACATGCAG. *AtU6* and *Hc5S* rRNA were used as internal reference for miR172e expression analysis. For the expression of other genes from various samples in *Arabidopsis*, cDNA was synthesized using cDNA Synthesis SuperMix (TianGen, Beijing, China) following the manufacturer’s instructions, and *AtActin* was used as the internal control, and the hygromycin gene was applied for the internal control in *Nicotiana benthamiana* transient expression assays.

Missing details of the stem-loop method for HcmiR172e expression, including the primer information, have been supplemented.

A correction has been made to Materials and Methods 4.5. Treatment of Transgenic *Arabidopsis*. The updates are as follows:

For the construction of HcmiR172e-OE *Arabidopsis*, *HcmiR172e* precursor was transformed into *Arabidopsis* (Col-0) via the floral dip method mediated by *Agrobacterium tumefaciens* (GV3101) containing pCAMBIA2300-*pre-HcmiR172e*. And then, homozygous, single-copy and highly expressed T_2_ transgenic lines were further screened for subsequent experiments [43].

A correction has been made to Discussion 3.1. HcmiR172e Targeted *HcTOE3* and Negatively Regulated Stress Tolerance. The full paragraph is updated as follows:

In our study, expression of HcmiR172e decreased, while *HcTOE3* increased in *H. caspica* under salt, drought and freezing stress. Because HcmiR172e negatively regulated *HcTOE3* via cleavage, the reduced HcmiR172e level led to suppressed cleavage and consequent accumulation of *HcTOE3*, which may regulate stress tolerance. This pattern contrasted with that in tomato [15], where stress up-regulated miR172 to reduce target gene levels against abiotic stresses. This discrepancy may be attributed to species specificity in downstream regulatory pathways. We confirmed the response of the HcmiR172e/*HcTOE3* module to salt, drought and freezing stress in *Arabidopsis*.

A correction has been made to Discussion 3.2. Antagonistic Regulation of the HcmiR172e/*HcTOE3* Module in Physiological and Molecular Responses to Salt, Drought and Freezing Stresses. The updates are as follows:

The HcmiR172e/*HcTOE3* module may be involved in ABA-dependent signaling by the addition of exogenous ABA for the observations of HcmiR172e and *HcTOE3* transgenic *Arabidopsis* at the stage of seed germination. Under exogenous ABA treatment, HcmiR172e-OE *Arabidopsis* showed concentration-dependent reductions in germination rate, cotyledon greening rate and root length, while *HcTOE3*-OE *Arabidopsis* maintained stable growth performance comparable to WT.

A correction has been made to Discussion 3.3. Regulatory Mechanism of the HcmiR172e/*HcTOE3* Module. The updates are as follows:

Integrating the above findings, the HcmiR172e/*HcTOE3* module was proposed to participate in ROS scavenging, osmotic adjustment and response to ABA to maintain cellular homeostasis, expanding our understanding on how halophytes coordinated multi-stress responses (Figure 8).

Overlong mechanistic speculation in the original text has been condensed into a concise and clear summary.

In these corrections, the unnecessary citations [3,4,27–29,31,46–52,56] have been removed, and the order of some references has also been adjusted accordingly.

Altogether, all authors state that the scientific conclusions are unaffected. These corrections were approved by the Academic Editor. The original publication has also been updated.
